# Does Smoking Cannabis Increase the Risk of Barrett’s Esophagus?

**DOI:** 10.7759/cureus.6913

**Published:** 2020-02-07

**Authors:** Joshua Levy, Keith Buhl, Christopher Fernandez, Jayaram Kumaraswamy

**Affiliations:** 1 Medicine, Philadelphia College of Osteopathic Medicine, Philadelphia, USA; 2 Gastroenterology, Nazareth Hospital, Philadelphia, USA; 3 Internal Medicine, Nazareth Hospital, Philadelphia, USA; 4 Internal Medicine, Philadelphia College of Osteopathic Medicine, Philadelphia, USA

**Keywords:** cannabis, barrett’s esophagus, esophageal metaplasia

## Abstract

Millions of Americans smoke cannabis every day. With the recent legalization of cannabis in many states, the number of Americans who smoke cannabis is expected to climb even higher. This case report presents a chronic cannabis smoker who developed severe Barrett’s esophagus at a young age. A 41-year-old African American male presented with an exacerbation of nausea and vomiting. The patient reported that he smoked cannabis two to three times daily for the past 20 years. Upper endoscopy and subsequent histology analysis displayed long-segment Barrett’s esophagus indefinite for dysplasia. The patient was encouraged to cease cannabis use and have a follow-up endoscopy in 3-6 months. Barrett’s esophagus is rare in African Americans; however, with the increase in the prevalence of cannabis smoking, endoscopic surveillance guidelines may need to be modified to include younger African Americans who chronically smoke cannabis.

## Introduction

Cannabis use has been documented for millennia, and it is sometimes suggested as a safer alternative to cigarette smoking [1]. Its use is climbing exponentially with the recent legalization of cannabis in many states. Chronic cannabis use may lead to nausea, vomiting, and abdominal pain such as in cyclic vomiting syndrome [2]. Despite the well-known gastrointestinal side effects of chronic cannabis use, there have been no reported cases of Barrett’s esophagus with chronic cannabis use. 

## Case presentation

**Figure 1:**
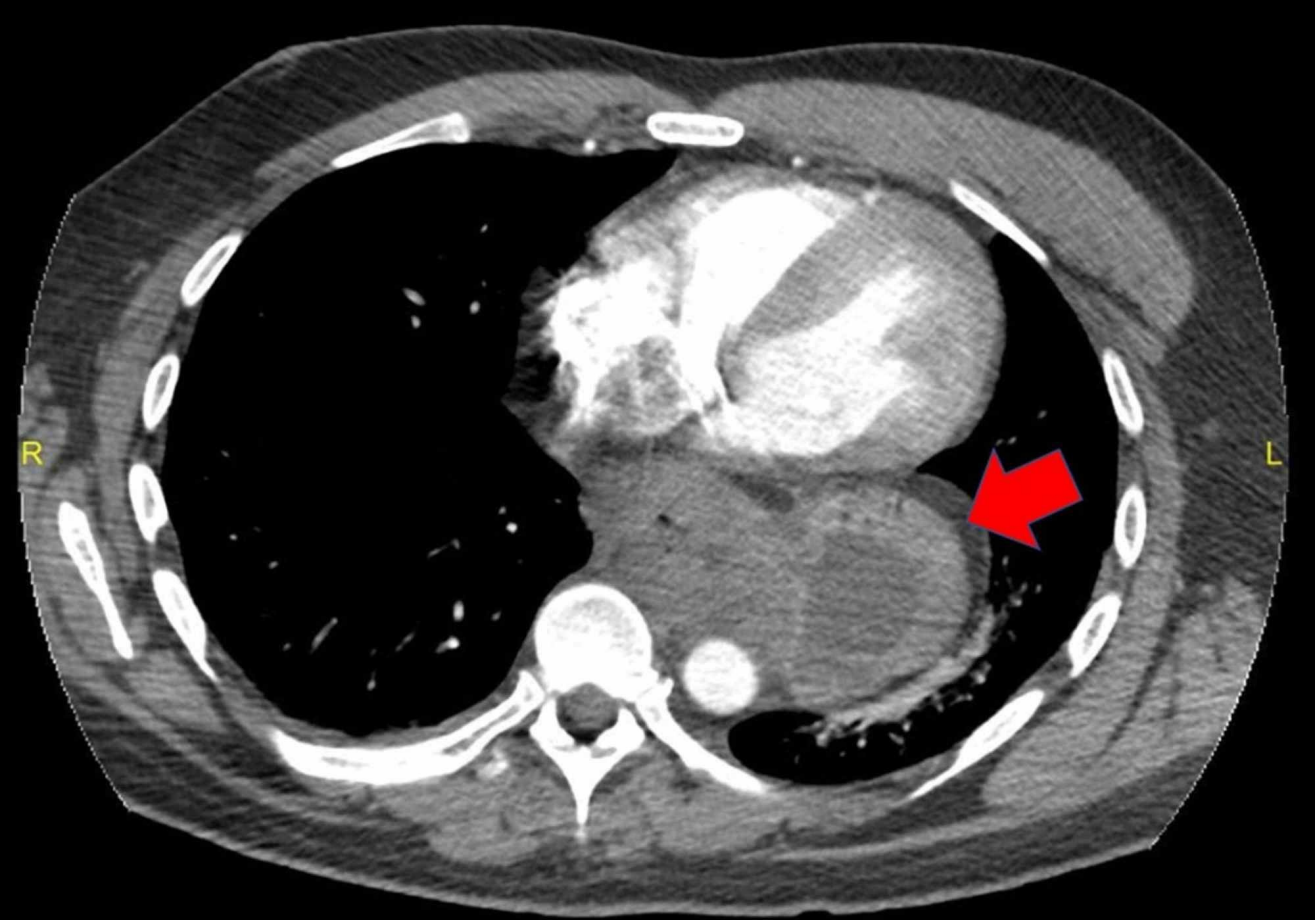
Contrast-enhanced computed tomography showed a large hiatal hernia with marked thickening of the distal esophagus and gastroesophageal junction

**Figure 2:**
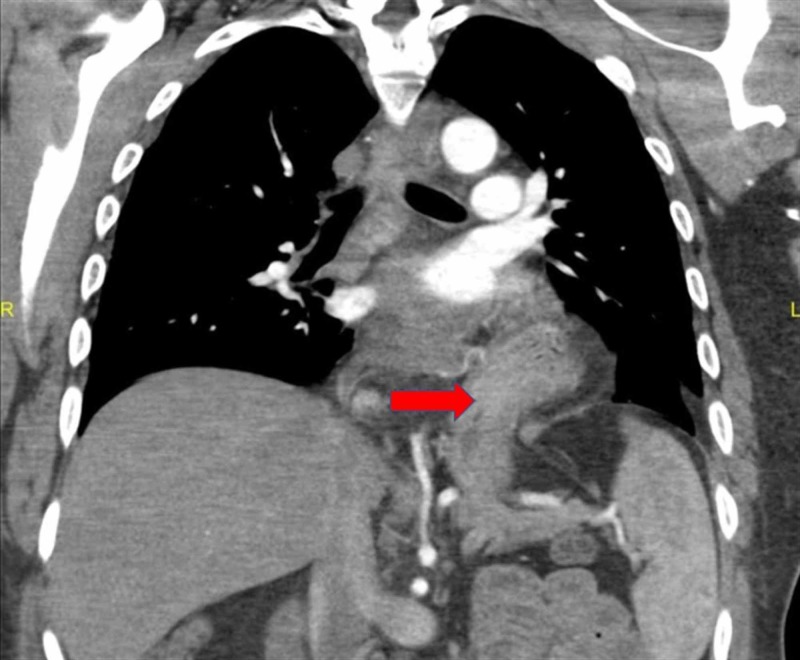
Contrast-enhanced computed tomography showed a large hiatal hernia with marked thickening of the distal esophagus and gastroesophageal junction

**Figure 3:**
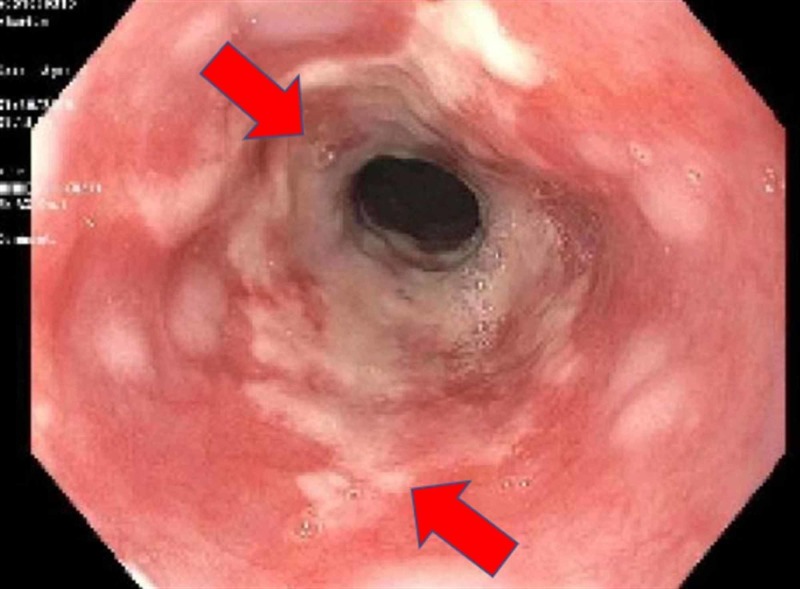
Diffuse salmon-colored mucosa and nodularity from 20cm to the esophagogastric junction at 30cm

**Figure 4:**
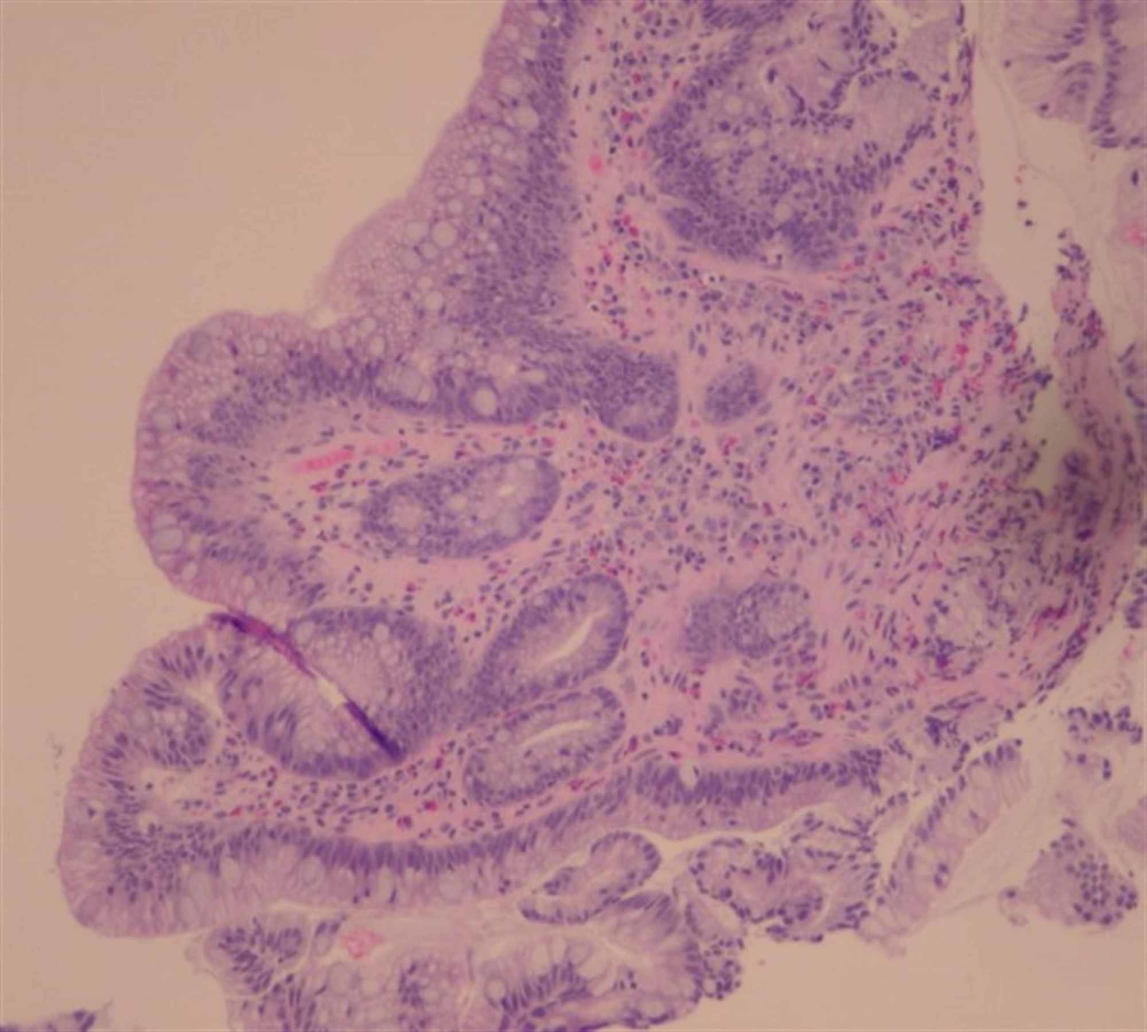
Columnar mucosa with intestinal cell metaplasia and glandular atypia indefinite for dysplasia

A 41-year-old African American male presented to the emergency room of a large academic urban hospital with abdominal pain and exacerbation of nausea and vomiting, which he had experienced episodically over the last two years. Up until this time, he was successful in using hot showers to relieve the nausea and vomiting. He denied abdominal distention, fever, or constipation. He smoked cannabis two to three times daily over the past 20 years. Vital signs were stable, and his physical exam was unremarkable with a normal body mass index. Contrast-enhanced computed tomography (CT scan) showed a large hiatal hernia with marked thickening of the distal esophagus and gastroesophageal junction (Figure 1, 2). No other abnormalities were noted. Upper endoscopy showed diffuse salmon-colored mucosa and nodularity from 20cm to the esophagogastric junction at 30cm, as shown in Figure 3. Histology showed columnar mucosa with intestinal cell metaplasia and glandular atypia indefinite for dysplasia, as seen in Figure 4. The patient’s condition improved, and he was discharged on a proton-pump inhibitor (PPI) and encouraged to cease cannabis use. He was recommended to have a follow-up endoscopy in 3-6 months. 

## Discussion

Cannabis comes from the *Cannabis sativa* or *Cannabis indica* plant. Tetrahydrocannabinol (THC) is the primary chemical through which Cannabis exerts its effect. Documentation of cannabis use dates to Asia circa 500 BC. Ancient cultures used cannabis primarily as an anesthetic and analgesic. Cannabis became a popular recreational drug in the United States in the early 1900s when it was brought over via Mexican immigrants escaping the Mexican revolution. According to the Centers for Disease Control and Prevention (CDC), as of 2015, 22.2 million individuals smoke cannabis every day [3]. Since cannabis use has increased dramatically, more research has been devoted to the drug and its adverse reactions. Nausea, vomiting, and cyclic vomiting syndrome are well-described complications of chronic cannabis use [4]. There are no case reports in the literature of the habitual use of cannabis and the subsequent diagnosis of Barrett’s esophagus. 

The word esophagus is derived from the Greek word *oisophagos, *which means gullet. The root *oisein *means “to carry” while the word *phagein *means “to eat.” The esophagus is a fibromuscular tube that enables food to pass from the oral cavity to the stomach. It is normally lined by stratified squamous epithelial cells. Stratified squamous epithelium is found on organs that are exposed to the outside environment, such as the anus and vagina. The stratified squamous epithelium of the esophagus provides protection and stability. An intact layer of the stratified squamous epithelium is vital to enable food to travel safely from the oral cavity to the stomach.

Barrett’s esophagus is a disease in which the normal squamous epithelium is replaced by mucus-secreting metaplastic columnar epithelium that usually lines the stomach. A cause of Barrett’s esophagus is gastroesophageal reflux disease (GERD). The constant reflux of acidic juices from the stomach into the esophagus places stress on the normal squamous epithelium of the esophagus. In order to better address the stress caused by the acidic juices, the squamous epithelium undergoes metaplasia to mucus-secreting columnar epithelium. Barrett’s esophagus is often asymptomatic and goes unrecognized. Occasionally individuals with Barrett’s esophagus can develop esophageal ulcers and other complications. Approximately 0.1-3% of individuals with Barrett’s esophagus develop adenocarcinoma.

Screening and early detection of Barrett’s esophagus are vital in preventing the feared complication of esophageal adenocarcinoma. Current screening recommendations focus on individuals with multiple risk factors for adenocarcinoma, such as patients who are obese, smoke, or have a family history of Barrett’s esophagus or esophageal adenocarcinoma. If dysplastic Barrett’s esophagus is detected, treatment options such as radiofrequency ablation and photodynamic therapy can be used to prevent the progression to adenocarcinoma. 

Barrett’s esophagus is most common in Caucasian men over the age of 55. The lowest prevalence of Barrett’s esophagus is in African Americans. The two most common types of Barrett’s esophagus are short and long-segment Barrett’s. The short-segment involves less than 3cm of Barrett’s mucosa, while the long-segment involves 4-10cm of Barrett’s mucosa. Short-segment Barrett’s is much more prevalent than long-segment Barrett’s. Because Barrett’s esophagus most commonly presents in Caucasian men over the age of 55, screening is primarily aimed at this population [5].

Many risk factors have been implicated in the development of Barrett’s esophagus. GERD, obesity, and smoking are some of the well-known risk factors. Our culture views cannabis as a safer alternative to other drugs. The perceived risk of using cannabis is at an all-time low, and the prevalence of cannabis use is at an all-time high. We suggest that chronic cannabis use may be a significant risk factor for the development of Barrett’s esophagus at a younger age and in populations where Barret’s is not usually prevalent. Our patient, a young African American male, smoked cannabis regularly for 20 years and presented with extensive long-segment Barrett’s esophagus. If cannabis is a contributing factor to the development of Barrett’s esophagus, it is important that patients and physicians are aware of this so that it can be screened for and addressed accordingly.

## Conclusions

Although other factors such as GERD could have contributed to the patient’s extensive Barrett’s esophagus, he had no typical reflux symptoms. It is hard to ignore the extensive use of cannabis as a possible contributing etiology of the disease process. African Americans have a higher prevalence of cannabis abuse than Caucasians and can thus be more at risk for the development of Barrett’s esophagus from smoking Cannabis. Although Barrett’s esophagus is unusual in African Americans, perhaps endoscopic surveillance needs to be modified in the future for African American patients who chronically use cannabis. 
